# Mucosal Alpha-Papillomavirus (HPV89) in a rare skin lesion

**DOI:** 10.1186/s12985-015-0336-y

**Published:** 2015-07-07

**Authors:** Francesca Paolini, Carlo Cota, Ada Amantea, Gianfranca Curzio, Aldo Venuti

**Affiliations:** HPV-UNIT Laboratory of Virology, Regina Elena National Cancer Institute, via Chianesi 53, 00144 Rome, Italy; Laboratory of Cutaneous Histopathology, San Gallicano Dermatologic Institute, Rome, Italy

**Keywords:** HPV 89, Syringocystoadenoma papilliferum, Apocrine acrosyringeal keratosis

## Abstract

**Background:**

Apocrine acrosyringeal keratosis is a rare skin lesion showing a unique benign keratotic lesion associated with syringocystoadenoma papilliferum. It is characterized by an exophytic proliferation of the epidermis with two distinct keratinocytic structures: a) columns of hyperkeratotic mass surrounded by acanthotic epidermis and b) papillated and/or cystic invaginations typical of syringocystoadenoma papilliferum. No causative agents were reported.

**Findings:**

The present report describes a typical case of apocrine acrosyringeal keratosis localized in the right retro-auricular area of 57-year-old man in which the presence of HPV was evaluated. PCR analysis and direct sequencing revealed the presence of HPV 89. The presence of this low risk mucosal HPV in a skin localization was never reported as well as in association with this rare tumor. Furthermore rolling circle amplification, RT-PCR and in situ hybridization confirmed the presence of a transcriptionally active HPV 89.

**Conclusions:**

Taken together our results suggest that HPV89 plays a role in apocrine acrosyringeal keratosis with syringocystoadenoma papilliferum, in consideration of the documented biological activity of the virus. The association of low risk mucosal HPV infection with this skin lesion opens new perspectives in its clinical management. Further studies on samples from other patients are needed to confirm this association.

## Findings

Human papillomaviruses (HPVs) can be considered etiological agents of invasive cervical, anogenital, and oropharyngeal, in particular tonsil, cancers [1, 2]. Currently, more than 180 HPV types have been fully characterized; the majority clusters into 3 genera: *Alphapapillomavirus* (alpha-HPV), mainly isolated from genital lesions; *Betapapillomavirus* (beta-HPV) and *Gammapapillomavirus* (gamma-HPV), the latter two genera predominantly isolated from skin [3].

The tissue tropism of a group of viruses is an important characteristic to understand how viruses evolve in ecological niches and induce pathogenic consequences in their hosts. In line with this assumption persistent infections by mucosal high-risk oncogenic alpha HPV types such as HPV type 16 are associated with some oral and/or oropharyngeal cancers [2, 4]. However, recent reports indicate that the oral cavity contains a wide spectrum of known and novel HPV types that phylogenetically cluster into the beta- and gamma-HPV genera, which were previously considered to be nearly exclusively skin types [5]. The difference in the spectrum of HPV types detected in the oral cavity and exfoliated cervicovaginal cells has significant implications for our understanding of the anatomic tissue tropism, the evolution of HPVs, and the epidemiological association of HPV with oral and skin neoplasia.

On the contrary, mucosal types have been detected in skin lesions and in particular HPV 16 was consistently detected in periungueal carcinoma with Bowenoid features [6]. Rare detection of other mucosal types has been reported suggesting that HPV 16 is the only mucosal type that possesses the ability not only to expand into multiple human epithelial niches but also to cause lesions in mucosal and cutaneous localizations [7].

**Fig. 1:**
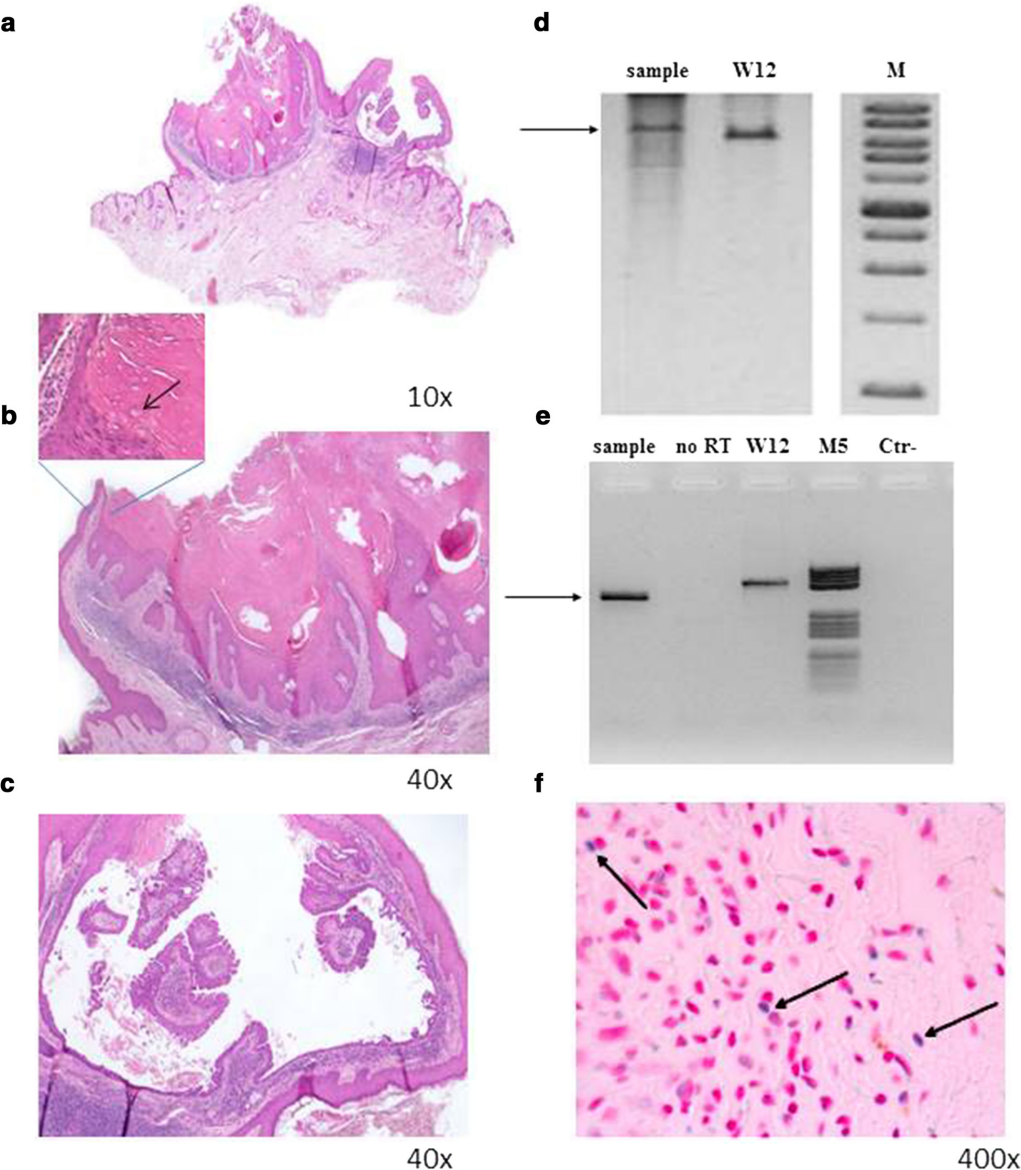
Histological findings and HPV detection. **a** Apocrine acrosyringeal keratosis associated with syringocystoadenoma papilliferum. Higher magnification of a warty-like area of apocrine acrosyringeal keratosis (**b**) and of a cystic area of syringocystoadenoma papilliferum (**c**). The arrow in the inset indicates a cell with perinuclear halo suggestive of a possible HPV infection. **d** RCA. Genomic DNA extracted and purified from the paraffin embedded sample was amplified with TempliPhi Amplification Kit (Amersham Biosciences, Milan, Italy) according to the manufacturer's instruction except an higher (450 mM) concentration of nucleotides. To resolve the concatemers, RCA products were digested with BamHI, (Invitrogen-Life Technologies, Monza, Italy), for 3 h at 37 °C in a total volume of 20 μl. Digestion products were resolved by 0.8 % agarose gel electrophoresis. W12 is a cell line containing episomal HPV16. M is a Xlarge DNA Ladder (GeneDirex,Rome, Italy). The arrow indicates the amplified band of about 8000 bp. **e** RT-PCR. Total RNA extracted and purified from the paraffin embedded sample was subjected to retro-transcription and nested PCR with degenerate primers. Amplified products were resolved in ethidium bromide stained agarose gel. W12 is a cell line expressing HPV16 mRNA. No RT is a control in which reverse transcriptase was omitted to exclude the presence of contaminating genomic DNA. M5 is a DNA Molecular Weight Marker V (Roche, Milan, Italy). The arrow indicates the amplified products. **f** ISH: In situ hybridization was performed with ZytoFast kit (Bioptica, Milan, Italy). Specific probes for HPV 89 were prepared by the asymmetric PCR [16] with consensus primers. The arrows indicate cells with HPV positive green/blue stained nuclei. Since consecutive sections were not available, the corresponding localization in H/E preparation cannot be displayed

This study was approved by the local Ethical Committee (Prot.n. CE/312/05). A 57-year-old man was showing a lesion in the right retro-auricular area that was diagnosed as apocrine acrosyringeal keratosis. Anything relevant to this pathology was in the clinical history of the patient and in particular no evidence of immunosuppression. The lesion was exophytic and revealed two different components: a well-developed syringocystoadenoma papilliferum (SCAP) with free floating papillae and a more verrucous proliferative area resembling a wart (Fig. 1a,b,c). This wart-like area showed acanthosis and papillomatosis of the epitelium with focal trichilemmal keratinization; in some areas the presence of hypergranulosis, columns of parakeratosis and perinuclear halo were suggestive of a possible HPV infection (Fig. 1b high magnification inset). Therefore, we decided to ascertain the presence of HPV by different methods.

Sensitive PCR with degenerate primers followed by direct sequencing was utilized to detect a broad spectrum of HPV types [5, 8]. Rolling circle amplification (RCA), a methodology that amplifies circular DNA [9], reverse transcriptase (RT)-PCR followed by direct sequencing and in situ hybridization (ISH) were also employed to define physical status and mRNA transcription [10].

**Fig. 2:**
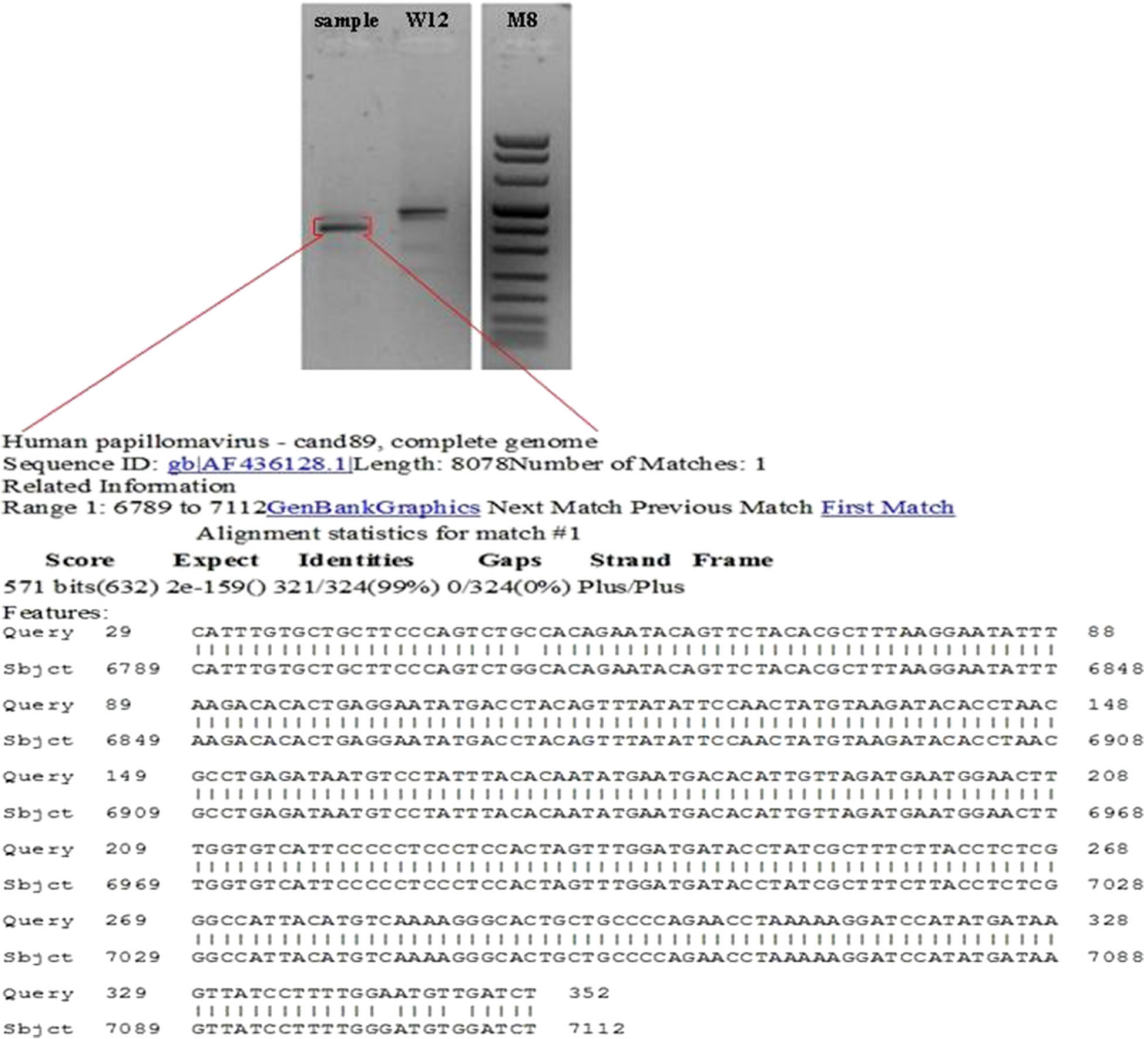
HPV detection and typing. DNA from clinical sample was analysed for HPV detection by PCR with CP degenerate primers that amplify a broad spectrum of HPVs. PCR conditions were 3 mM MgCl2, 200 μM dNTPs, 0.5 μM for each primer and 2.5 U of Platinum TaqDNA polymerase (Life technologies, Milan, Italy) in a final reaction volume of 50 μl. Amplified products were sequenced in an automatic apparatus (Genechron Biogen, Rome, Italy) and sequence analyses were performed using BLAST program (http://www.ncbi.nlm.nih.gov/BLAST). W12 is a cell line containing episomal HPV16 utilized as positive control. M, molecular weight marker VIII (Roche, Milan, Italy)

PCR analysis demonstrated the presence of an amplified band that upon direct sequencing revealed a nucleotide sequence with 98 % homology to that of HPV 89 (Fig. 2). RCA showed a band of about 8000 bp after digestion with single cutting BamHI restriction enzyme, indicating the presence of episomal virus. This band was higher than that of episomal HPV16 extracted from W12 control cells because these two viruses are different in genome length (Fig. 1d). To confirm that RCA product was from HPV 89, RCA products were digested with another single cutting enzyme (KpnI), amplified with CP degenerate primers and subjected to direct sequencing that confirmed the presence of HPV type 89.

HPV mRNA expression was detected by reverse transcriptase–PCR (Fig. 1e). Total RNA was extracted from dewaxed sections according to standard procedures that allow the recovering of PCR-grade RNA [11, 12]. In addition, the quality of RNA was preserved by maintaining the biopsy in buffered formalin for a short period of time. cDNA was retro-transcribed by a commercial kit (Superscript Reverse Transcriptase, Life Technology, USA). CP primers consisting of two sets of degenerate primers located in the late L1 ORF were utilized to amplify HPV sequences from cDNA [5, 8]. The annealing sites of these primers have different positions in the genome of different HPV types. The CP65-CP70 primer set and the nested CP66-CP69 primer set amplify products ranging from 452- to 467-bp and from 374- to 389-bp, respectively. Indeed, the position of HPV89 amplified band is slightly different from that of HPV16 form W12 cells (Fig. 1e). The amplified bands were excised from the agarose gel and subjected to direct sequencing that confirmed the presence of HPV89 L1 sequences.

Since no cloned HPV89 was available, viral load was measured in real time-PCR respect to two different standard curves: SiHa cells with one integrated copy of HPV16 and a plasmid carrying HPV16 full genome (mimicking episomal HPV). Briefly, real time PCR was performed with Kapa SYBR Green Master Mix (KAPA-biosystems , Milan, Italy) in i-Cycler apparatus (BioRad Laboratories Inc., Milan, Italy) according to manufacturer's instructions. Amplifications of L1 and β-globin gene were performed in a 20 μl volume containing 2× Kapa SYBR Fast qPCR Master Mix, 10 μM of each primer and 20 ng of genomic DNA, using the following protocol for 40 cycles: 3 s denaturation at 95 °C, annealing and extension at 60 °C for 30 s, with the initial denaturation at 95 °C for 3 min. Viral load was normalized by β-globin gene and calculated as 0.73 copy/genome equivalent. Finally, ISH revealed a HPV positive nuclear signal confirming data of RCA about the presence of episomal HPV 89 (Fig. 1f).

In the present report, we bring evidence that transcriptionally active low risk HPV 89 was present in apocrine acrosyringeal keratosis, a unique benign keratotic lesion associated with syringocystoadenoma papilliferum described on 2000 by Khishimoto et al. [13].

It is characterized by an exophytic proliferation of the epidermis with two distinct keratinocytic structures: a) columns of hyperkeratotic mass with swollen corneocytes containing keratohyalin granules surrounded by acanthotic epidermis and b) papillated and/or cystic invaginations, typical of SCAP. This tumor is rarely observed and, at the best of our knowledge, only one case is reported in the English literature. Khishimoto et al. made the hypothesis of HPV involvement but failed to detect any HPV by immunohistochemistry [13]. It is to note that they utilized a low sensitive assay to identify the presence of HPV.

On the contrary, Carlson and co-workers reported the presence of alpha and beta HPV DNA in cases of syringocystadenoma papilliferum associated with nevus sebaceus, a complex hamartoma of the skin with epithelial and dermal components, often associated with secondary neoplasms as trichoblastoma, basal cell carcinoma and syringocystadenoma papilliferum [14].

The presence of this low risk mucosal HPV in a skin localization was never reported as well as in association with this rare tumor. HPV 89 was recently associated with a long duration of viral persistence in limited case studies suggesting some peculiar characteristic of this HPV [15]. Taken together our results suggest that HPV89 plays a role in this rare pathology. In favor of this hypothesis is the presence of episomal DNA and of viral mRNA for L1 capsid protein (productive infection) as well as a histology suggestive of HPV infection, whereas against this hypothesis is the low copy number. However, results from ISH may suggest that in few cells the HPV89 is in high copy number compatible with viral expression.

In alternative, the virus presence, even if transcriptionally active, may represent only a reactivation of latent infection during the lesion development.

Nevertheless, the ascertained presence of mucosal papillomavirus in skin locations reinforces the hypothesis that a single type of HPV may have a different tropism in comparison with other members of the same species suggesting that genotyping classification may not always reflect the biological behavior of the HPVs. The role of symbiotic HPV infections, their relationship with the host (e.g. commensal) and the emergence of their pathogenic potential remain to be better understood.

In conclusion, apocrine acrosyringeal keratosis with syringocystoadenoma papilliferum can be associated with HPV infection. Although conclusive data cannot extrapolated from a single case, nevertheless the possibility of an association of this rare skin pathology with an Alfa papillomavirus exists in consideration of the documented activity of the virus. Further studies on samples from other patients are needed to confirm this association.
